# Chopper-modulated gas chromatography electroantennography enabled using high-temperature MEMS flow control device

**DOI:** 10.1038/micronano.2017.62

**Published:** 2017-12-18

**Authors:** Ming-Da Zhou, Muhammad Akbar, Andrew J. Myrick, Yiqiu Xia, Waleed J. Khan, Xiang Gao, Thomas C. Baker, Si-Yang Zheng

**Affiliations:** 1 Micro & Nano Integrated Biosystem (MINIBio) Laboratory, Department of Biomedical Engineering, The Pennsylvania State University, University Park, PA 16802, USA; 2 Materials Research Institute, The Pennsylvania State University, University Park, PA 16802, USA; 3 Department of Entomology, The Pennsylvania State University, University Park, PA 16802, USA; 4 Department of Electrical Engineering, The Pennsylvania State University, University Park, PA 16802, USA

**Keywords:** electroantennography, gas chromatography, MEMS flow control device, microvalve

## Abstract

We report the design, fabrication and characterization of a microelectromechanical systems (MEMS) flow control device for gas chromatography (GC) with the capability of sustaining high-temperature environments. We further demonstrate the use of this new device in a novel MEMS chopper-modulated gas chromatography-electroantennography (MEMS-GC-EAG) system to identify specific volatile organic compounds (VOCs) at extremely low concentrations. The device integrates four pneumatically actuated microvalves constructed via thermocompression bonding of the polyimide membrane between two glass substrates with microstructures. The overall size of the device is 32 mm×32 mm, and it is packaged in a 50 mm×50 mm aluminum housing that provides access to the fluidic connections and allows thermal control. The characterization reveals that each microvalve in the flow control chip provides an ON to OFF ratio as high as 1000:1. The device can operate reliably for more than 1 million switching cycles at a working temperature of 300 °C. Using the MEMS-GC-EAG system, we demonstrate the successful detection of *cis*-11-hexadecenal with a concentration as low as 1 pg at a demodulation frequency of 2 Hz by using an antenna harvested from the male *Helicoverpa Virescens* moth. In addition, 1 μg of a green leafy volatile (GLV) is barely detected using the conventional GC-EAG, while MEMS-GC-EAG can readily detect the same amount of GLV, with an improvement in the signal-to-noise ratio (SNR) of ~22 times. We expect that the flow control device presented in this report will allow researchers to explore new applications and make new discoveries in entomology and other fields that require high-temperature flow control at the microscale.

## Introduction

Gas chromatography (GC) is one of the most widely used technologies around the world for the identification of volatile organic compounds (VOCs). Various research disciplines, including environmental monitoring, biomedical diagnostics, entomology, fossil fuel exploration and food processing, have benefitted from GC technology^1–5^. Microfabrication technology has enabled important advancements in GC-related technologies. For example, there has been increasing interest in the development of miniaturized GC (μGC) systems, which can provide rapid diagnosis of VOCs with low cost and low power consumption. A standalone μGC is realized using three miniaturized components: a microinjector/preconcentrator for sample introduction^6–8^, a microcolumn for VOC separation^9,10^ and a detector for identifying the separated compounds^11–13^. Ever since its inception^14^, the majority of research in μGC has been directed toward the fabrication of the above-mentioned basic components^15^. Less effort has been made toward the fabrication of other necessary μGC components, such as microvalves and micropumps, which are used extensively for fluidic routing and to provide gas flow. Usually, a μGC system is developed using commercially available valves and pumps^16–18^, and few research milestones have been accomplished toward microvalve miniaturization^14,19^. In one example, Terry *et al*^20^ reported a magnetically actuated microvalve for sample injection in their seminal work on μGC development. Their microvalve comprised a nickel membrane, silicon seat and a solenoid actuator and plunger assembly. Similarly, Dziuban *et al*^21^ presented a pneumatically actuated microvalve based on a polyimide membrane. The microvalve was 3.5 cm×3.5 cm×0.25 cm in size and was employed to inject sample volumes of approximately 12 μL. Kinda *et al*^14^ presented a microinjector with six low-leak microvalves made from polyetheretherketone membranes on silicon substrates. The overall dimensions of their microinjector were 7 mm×3 mm×1.2 mm, and it allowed the injection of a 250 nl sample volume. It is evident that the majority of microvalve designs rely on the actuation of a flexible membrane to control the flow direction. The actuation of a membrane can be achieved piezoelectrically^22^, electrostatically^23^, electromagnetically^20^, pneumatically^14^ or thermopneumatically^19^. Furthermore, these microvalves have been employed only as injection units for sample introduction and have not been demonstrated to operate in high-working-temperature environments. The use of microvalves for high-temperature GC operation (up to 300 °C) is therefore still largely under development. The implementation of devices of this type would benefit the future use of GC-based VOC analysis at the microscale level.

A major workhorse of entomology and chemical ecology research is the electroantennogram (EAG), which detects volatile odorants from the electrical response of insect antennae. When coupled with GC and/or mass spectrometry, the EAG can precisely identify the specific compounds sensed by an insect. Ever since its inception^24^, the GC-EAG technique has been essential and one of the most widely used technologies in chemical isolation and identification studies around the world for discovering new attractant blends of insect pheromones and host volatile attractants from natural sources. However, the conventional EAG is both noisy and orders of magnitude less sensitive than the ability of an insect to sense and respond to odorants^25^. Behaviorally active trace components, with regard to their molecular abundance, are often missed by conventional GC-EAG analysis because of the small background-noise-level depolarization evoked on an EAG. This is due to the small number of receptor neurons on the antennae devoted to their detection^26^. Thus, while EAG detection is well established in chemical ecology, it also suffers from a major bottleneck that limits the discovery, understanding and control of insect behavior^27^. New advances are needed to improve the signal-to-noise capabilities of this valuable technique. We propose a microelectromechanical systems (MEMS)-enabled chopper-modulated GC-EAG (MEMS-GC-EAG), which can deliver enhanced signal-to-noise ratio (SNR) detection abilities for trace biologically active organic components that otherwise would be missed.

**Figure 1:**
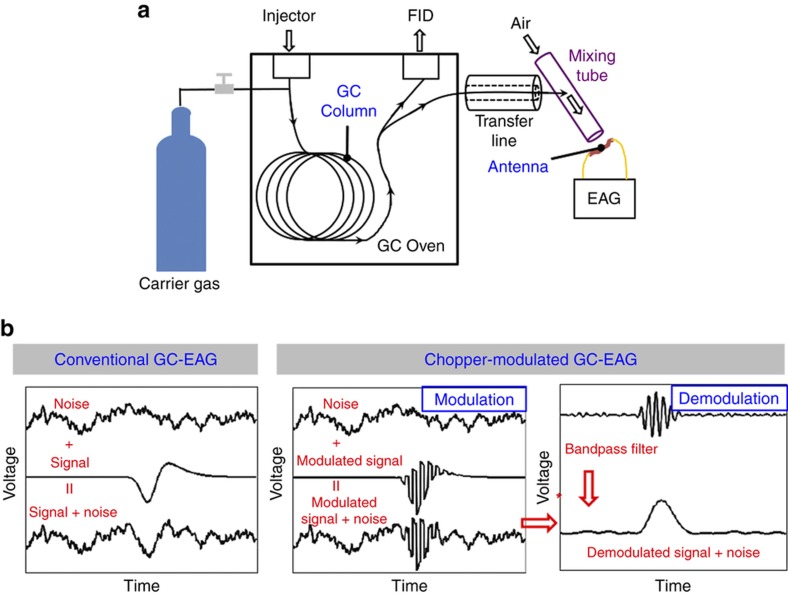
Principle of chopper-modulated gas chromatography-electroantennography (GC-EAG). (**a**) Operation of conventional GC-EAG. (**b**) In chopper-modulated GC-EAG, modulation of the raw signal, followed by signal filtration and demodulation, allows the retrieval of weak signals from a noisy environment. FID, flame ionization detector.

Signal chopping is an established technique for improving the SNR of signals buried in noise (typically 1/f-type noise). In optical detection, optical switches implemented using optical components (such as a chopper wheel, a piezoelectric phase modulator, an acousto-optical modulator or a photoelastic modulator) are used with lock-in-amplifiers to improve the signal SNR^28,29^. In chopper-stabilized amplifiers, the input waveform is rapidly multiplied by a square wave using switches and then amplified and demodulated by multiplying the amplifier output by another square wave of the same frequency^30^. Similar to the optical and electrical implementation of signal chopping, the input waveform of a chopper-modulated GC-EAG is rapidly multiplied mechanically by a square wave using the MEMS flow control device, amplified and then demodulated by multiplying the amplifier output by another square wave of the same frequency (Figure 1). The EAG has a noise power spectral density that decreases with *f*^−2^. Consequently, the SNR is improved by shifting the signal energy to a higher frequency. This can be achieved via signal chopping, which shifts a significant portion of the signal energy to the chop frequency, where there is less noise. After demodulation via bandpass filtering, the noise outside the chopping frequency is removed, and the signal can be recovered with an improved SNR.

We previously proposed a chopper-modulated GC-EAG principle and demonstrated it using an effluent chopper prototype that was constructed from off-the-shelf components that were placed outside a GC oven^26,31^. The mechanical chopper in the prototype improved the SNR of the EAG but also inevitably induced mechanical noise. Herein, we report the development of a MEMS flow control device constructed using glass and a polyimide membrane, both of which are fully GC compatible. The device consists of a novel design that allows chopping of the input sample and feeds it simultaneously to both the EAG and a flame ionization detector (FID) for detailed analysis. The MEMS flow control chip is actuated pneumatically using four commercially available solenoid valves and can operate at temperatures as high as 300 °C. The flow control device resides inside the GC oven and enables the chopping of GC effluent without the induction of mechanical noise. We demonstrate that a pheromone concentration as low as 1 pg can be successfully detected using the MEMS-GC-EAG technique.

## Device architecture and operation

Three main structural criteria were considered while designing the flow control device. First, the dead volume needs to be well below 1 μL to minimize diffusion and enable sufficiently fast switching. The current design has a dead volume of 50 nl. Second, the chip needs to be able to run at high temperatures (up to 300 °C) for several hours^32^, which limits the choice of substrate and membrane material that can be used for fabrication of the device. Polyimide (working temperature: 360 °C), used as a membrane, and borosilicate glass (working temperature: 560 °C) were selected for this purpose. Third, low outgassing as well as minimum absorption is desired for all materials in contact with the column effluent. The materials that interface with the gas effluent in our design are glass, polyimide and graphite, all of which have been widely used in conventional GC.

**Figure 2:**
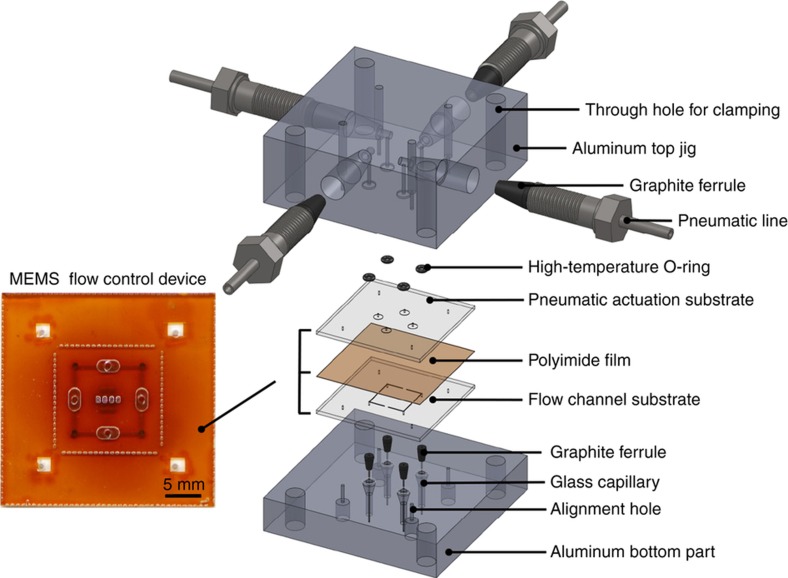
Three-dimensional exploded view showing the design of the microelectromechanical systems (MEMS) flow control device. The inset on the left is a picture of the fabricated MEMS flow control device (top view), which includes four microvalves with an oval shape, four microchannels and four inlet/outlet ports.

The flow control device is a microfluidic chip with four pneumatically controlled microvalves connecting the four ports with microfluidic channels, as shown in Figure 2. We selected pneumatic operation as the actuation mechanism for the microvalves because unlike electrostatic or piezoelectric actuation, pneumatic actuation does not require additional materials to be incorporated into the valve membrane. This enhances the reliability of the microvalve since the material incorporated into the membrane can be delaminated under high-working-temperature conditions. Furthermore, pneumatic actuation requires compressed air and vacuum lines. The former is a standard GC consumable, and the latter is typically available in GC labs.

**Figure 3:**
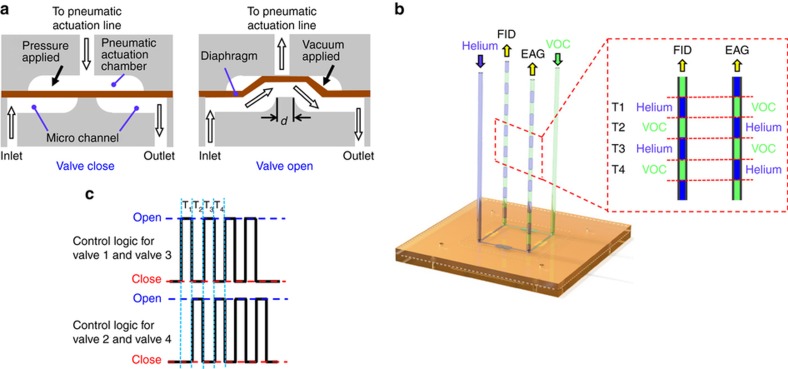
Design and operation of the microelectromechanical systems (MEMS) flow control device. (**a**) Schematic of the opening and closing of the microvalve under pneumatic control. Channel separation d is defined in the right figure. (**b**) Chopping of the effluent from the GC separation column. The four microvalves are labeled from 1 to 4 clockwise. By feeding the effluent of the GC separation column and helium gas into two diagonal inlets of the MEMS flow control device and applying the control logic to the valves continuously, the two diagonal outlets will output chopped GC effluents with a 180° phase shift. (**c**) The pneumatic control logic for chopping of the effluent from the GC separation column. EAG, electroantennography; FID, flame ionization detector; GC, gas chromatography; VOC, volatile organic compound.

When a positive pneumatic pressure is applied to the pneumatic actuation chamber, the membrane is pushed against the valve seat between two microfluidic channels, thereby closing the valve. When a vacuum is applied, the membrane in the actuation chamber is pulled away from the valve seat. The valve is now opened, and the gas is allowed to flow freely between the two fluidic ports connected by the microvalve, as shown in Figure 3a. The separation between two adjacent channels, *d*, is designed to be 500 μm. The VOC effluent from the GC separation column and the helium gas are fed through the two diagonally positioned fluidic ports, which serve as the inlets of the device. The outlets of the device are fed to the FID and the mixing tube of the EAG system, respectively, as shown in Figure 3b. The four microvalves work in such a way that the two nonadjacent valves always have the same open/close status, while the two adjacent ones always have opposite open/close statuses, as shown in Figure 3c. In this way, VOCs from the GC column are directed to the FID and EAG alternately at a frequency that is controlled by the switching of the solenoid valves. Thus, both the FID and EAG receive chopped VOC signals.

## Materials and methods

### Fabrication and assembly

The fabrication of the flow control device starts with the patterning and etching of structures on glass substrates. One glass has microfluidic flow channels and four ports and is referred to as the flow channel substrate. The channel is 250 μm wide and 100 μm deep, which is comparable to the internal diameter (i.d.) of small GC columns. The other glass has four etched chambers and four pneumatic control ports from the other side. Each chamber is 100 μm deep and has the shape of two 1.6 mm diameter half-circles separated by a square of 1.6 mm ×1.6 mm. This glass is referred to as the pneumatic actuation substrate. Both glass substrates were fabricated from 4 inch ×4 inch square Borofloat photomask blanks precoated with 110 nm-thick chrome and a 530 nm-thick photoresist AZ1518 (Telic Company, Valencia, CA, USA). Design patterns were transferred from a laser-plotted polyester film to the photomask blanks using a flood UV exposure system (Oriel, Irvine, CA, USA). After being developed in AZ developer (Clariant, Somerville, NJ, USA), the chrome layer was etched in CR-7 (Cyantek, Fremont, CA, USA). The flow channels and chambers were then etched using 49% hydrofluoric acid to a depth of ~100 μm. After an additional photoresist layer was coated, the photomask blanks were diced using a dicing saw (ADT 7100, Yokneam, Israel). Finally, the remaining photoresist and chrome were removed using acetone and CR-7, respectively.

The flow control device was constructed via thermocompression bonding of the polyimide membrane between two microfabricated glass substrates, as shown in the exploded view of Figure 2. To start the bonding process, the polyimide film (DuPont, Wilmington, DE, Kapton HN) was cut down to an appropriate size. Pure polymer film without any coating was selected to meet the requirements of high temperature and low gas adsorption during operation. It is notoriously difficult for polyimide film to form strong bonds with other materials^33–35^. To improve the bonding strength, the polyimide film was cleaned via ultrasonication in acetone, IPA, and deionized (DI) water and baked in a vacuum oven at 120 °C for 2 h after undergoing a blow-dry process. The glass chips were activated using piranha solution (95% H_2_SO_4_: 30% H_2_O_2_=3:1) and baked at 115 °C for 5 min on a hot plate^36^. The surface of the glass chips and that of the polyimide film were further activated together using oxygen plasma (200 mTorr O_2_, 100 W, 10 min). The bonding was performed using a custom-made bonding jig made with polished aluminum blocks (Supplementary Information and Supplementary Figure S1), which was designed to provide uniformly distributed and controlled pressure to the MEMS flow control chip with four corner screws (thread size: 10–32). The flow channel substrate, polyimide film and pneumatic actuation substrate were aligned together with the custom-made bonding jig, using the alignment holes and pins. The screws were tightened to 0.68 N m using a torque wrench, and the whole assembly was annealed at an elevated temperature (450 °C) inside a vacuum oven filled with inert N_2_ gas for 2 h and then allowed to cool down naturally afterward^37^. Excellent bonding strength and repeatability were achieved by following this process.

**Figure 4:**
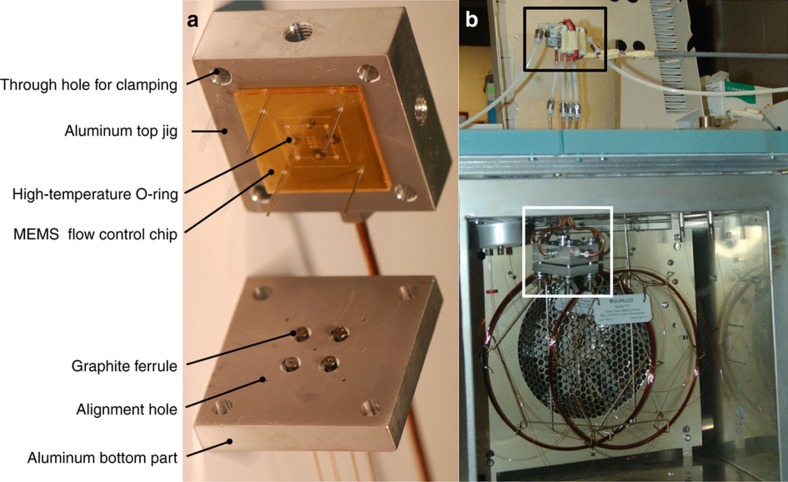
(**a**) Picture of the microelectromechanical systems (MEMS) flow control device in its housing. (**b**) Picture showing the location of the device (middle white box) inside the GC oven and the solenoid mini-valve array (top black box), which is used for pneumatic control outside the GC oven. GC, gas chromatography.

The packaging of the MEMS flow control device includes a precision computer numerical control (CNC)-machined aluminum housing that provides fluidic connections and temperature control to the device. The housing is composed of two aluminum parts: the top part provides a connection to pneumatic actuation lines, and the bottom one interfaces with the GC separation column, carrier gas, FID and EAG, as shown in Figure 4a. Aluminum was selected as the material for the housing because of its excellent thermal conductance and easy machining. The device is aligned to the housing through 0.8 mm diameter alignment holes in both device housings and clamped tightly using screws and nuts, as shown in Figure 4a. The pins are removed after the device is fully assembled. Pneumatic actuation lines are connected to the top part through four receiving ports and commercially available fittings (Valco Instruments Co. Inc., Houston, TX, USA) with high pressure ratings. Four high-temperature O-rings provide a seal between the top jig and the pneumatic control side of the device. The bottom part seals the GC column against the fluidic access ports of the device using graphite ferrules (Chromatography Research Supplies, Louisville, KY, USA). Because of the small footprint of the flow control device (32 mm×32 mm), the whole packaged device is miniaturized to ~50 mm×50 mm, which makes it easy to fit it inside the GC ovens. The heating element is a high-power resistor array that is bonded to the aluminum housing using high-temperature thermal epoxy (not shown in Figure 4a), which maintains the temperature of the valve at or slightly above that of the oven. Thermocouples (also not shown in Figure 4a) are mounted inside the oven and on the inner surfaces of both the top and bottom parts of the housing.

A LabVIEW program (National Instruments, Austin, TX, USA) was developed to provide the control logic. The temperature information from each thermocouple can be monitored in real-time and processed by the LabVIEW program using a dual proportional-integral-derivative control algorithm optimized with root-locus methods. The program also provides control signals to the heating units that are mounted on the outer surfaces of each aluminum part to drive the temperature of the flow control device to follow that of the oven.

To test the compliance of temperatures of the module to that of the GC oven, the oven temperature was increased from room temperature to 300 °C at a rate of 10 °C s^−1^, as shown in Supplementary Figure S2. The temperatures on both parts of the housing were measured and compared to the oven temperature. At this ramping rate, a power of approximately 30 W is required. Without a cooling mechanism, the temperature overshoot was ~2 °C. The system was also designed to limit the temperature difference (barely discernible) between the top and bottom of the housing.

The pneumatic actuation of the four microvalves was carried out using four high-speed solenoid mini-valves (SMC, Noblesville, IN, USA), which resided outside the GC oven, as shown by the black box in Figure 4b. The SMC microvalve has three ports (two inlets and one outlet) and a total power consumption of only 2 W. The two inlets of the solenoid valve are connected to compressed nitrogen and a vacuum pump, respectively, and the outlet, which is fed through the top of the oven wall, is connected to one of the pneumatic chambers of the flow control device. The electrical current needed to drive the solenoids is provided by a Darlington transistor array chip (TI ULN2003A). The timing of the solenoid valves is implemented using a computer running LabVIEW through a data acquisition board (NI USB-6212, National Instrument, Austin, TX, USA) with a resolution of 1 ms.

### Membrane deflection analysis

The deflection of the polyimide membrane under different applied pressures was simulated using the COMSOL software (COMSOL Inc., Burlington, MA, USA). The COMSOL simulation model was set up by selecting 3D as the space dimension, electromechanics as the physics interface and stationary from the preset study list. The dimensions of the membrane were the same as those of the actuation chamber in the flow control device (two 1.6 mm diameter half-circles separated by a square of 1.6 mm ×1.6 mm). Next, the boundary conditions for the membrane were defined by restricting the movement of the membrane edges to 0 in all directions. The properties of the polyimide membrane were specified in terms of Young’s modulus (3.1 GPa), density (1420 kg m^−3^) and Poisson’s ratio (0.34). Within the study node, the pressure was set as an auxiliary parameter, and its value ranged from 0 to 20 psi. The total and average displacement of the membrane was evaluated under different membrane thicknesses (25, 50 and 75 μm) and temperature conditions (23, 100, 200 and 300 °C).

### Leak rate characterization

The leak rate of the microvalves was characterized at room temperature using the setup shown in Supplementary Figure S3. Here, all four valves within the flow control device were closed by applying 25 psi of pneumatic actuation pressure. Helium was supplied through one of the inlet ports connected to the injector of a conventional GC system. Meanwhile, the port adjacent to the inlet was fed into a glass vial filled with DI water. The vial was held upside down, and any helium gas that leaked through the valve drained the same volume of water through the outlet of the vial. The volumetric leak rate of the valve was calculated using the following formula:

$$Q_{L}=\frac{W_{p}-W_{A}}{\rho \cdot t}$$

where *Q*_L_ is the volumetric leak rate, *W*_P_ is the total weight of the glass vial prior to testing, *W*_A_ is the total weight of the vial after testing, *ρ* is the density of the water and *t* is the time duration of the test.

### Switching speed and reliability characterization

The packaged device was installed inside the oven of a conventional Agilent 6890N GC system (Agilent, Santa Clara, CA, USA). A 30 m-long, 0.32 mm i.d. GC separation column (DB-5, Agilent, Santa Clara, CA, USA) was installed in the same oven, with its inlet connected to the injector and outlet connected to one of the inlet ports of the flow control device. One of the two outlet ports that are adjacent to the inlet in the device was connected to the FID of the GC system using a 40 cm-long, 0.32 mm i.d. GC capillary column. The rest of the fluidic ports were exposed to the atmosphere. The head pressure of the GC separation column was set at 15 psi. The positive pneumatic actuation pressure was provided by compressed nitrogen regulated at 25 psi, and the vacuum was provided using a continuously working vacuum pump.

During each trial, 1 μL of hexane was loaded into the GC separation column using a syringe through the injector of the GC system, which was operated in splitless injection mode. The four microvalves within the device followed the control logic generated by the LabVIEW program. The two microvalves adjacent to the inlet had opposite open/close statuses. The other two valves were kept closed at all times. As a result, the hexane effluent from the GC separation column was directed toward the FID and vented into the atmosphere in a chopping mode with a 62.5 ms interval. The signal from the FID was acquired with a 1 kHz sampling rate using a different LabVIEW program.

The reliability was investigated using the same experimental setup described for the switching time characterization; however, the temperature during the reliability test was kept at 300 °C. The oven temperature was increased to 300 °C and held for a sufficient period of time to allow the actual temperature of the device to reach 300 °C and stabilize. During each measurement, 1 μL of hexane was loaded into a 30 m-long GC column through the injector of the GC system in splitless mode.

### Pheromone detection

**Figure 5:**
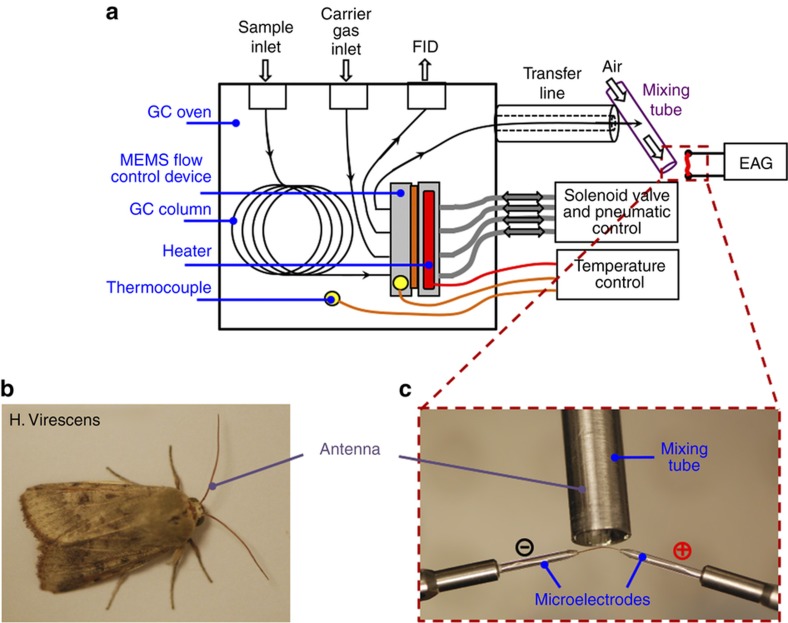
Detection of the pheromone *cis*-11-hexadecenal using microelectromechanical systems gas chromatography-electroantennography (MEMS-GC-EAG). (**a**) Overall testing system diagram. (**b**) Male *H. Virescens* moth, from which the antenna was harvested. (**c**) Close-up view showing the detailed arrangement of the antenna, mixing tube and microelectrodes. FID, flame ionization detector; H. Virescens, Helicoverpa Virescens.

Pheromones are VOCs secreted by insects and serve as chemical messengers, playing important roles in insect communication. The detection of pheromones by the MEMS-GC-EAG system was demonstrated using the modified Agilent 6890N GC system, as shown in Figure 5a. The flow control device was installed inside the oven. Chopped GC effluent from the device was delivered to the mixing tube outside the oven through a heated transfer line (Syntech, The Netherlands). A 30 m-long, 0.32 mm i.d. DB-1 GC separation column was installed in the GC system, with its outlet fed into the inlet port of the flow control device. Helium was supplied through the second inlet port of the device. The two ports that were adjacent to the inlet were fed into the mixing tube of the EAG system and the FID, respectively. Helium was employed as the carrier gas, with a constant flow rate of 7 mL min^−1^. The temperatures of the GC injector and FID were set at 300 and 220 °C, respectively. The temperature of the transfer line was set to 358 °C, which was much higher than the boiling temperature of the pheromone, to eliminate condensation at its outlet. Air, saturated with water vapor, was pumped into the mixing tube at a rate of 1.2 l min^−1^ to mix with the effluent delivered by the transfer line. The mixture was delivered to the antenna. The mixing tube cools and humidifies the effluent so that it does not damage the antenna. All four microvalves in the flow control device were switched in such a way that the adjacent ones always had opposite open/close statuses, while the opposite ones always had the same open/close status.

The major pheromone of the *Helicoverpa Virescens* (*H. Virescens*) moth, that is, *cis*-11-hexadecenal, was dissolved in hexane and diluted to different concentrations. The initial oven temperature of the GC system was 100 °C. After the temperature of the device reached the oven temperature and stabilized, the antenna was harvested from a male *H. Virescens* moth and mounted onto the microelectrodes after trimming off its tip, as shown in Figure 5b. The microelectrodes were prepared by pulling heated glass capillaries and filling them with 0.9% sodium chloride solution, which served to establish the electrical connection between the antenna and the silver-wire electrodes of the IDAC-2 data acquisition system (Syntech). The antenna was placed ~3 to 5 mm away from the outlet of the mixing tube, as shown in Figure 5c. The pheromone sample was then loaded into the GC separation column through the injector of the GC system in splitless injection mode. Following injection, the oven temperature was increased to 160 °C at a rate of 15 °C min^−1^ and then further increased to 200 °C at a rate of 10 °C min^−1^. Afterward, the oven temperature was maintained at 200 °C. Temperature programming was used to reduce the duration of the pheromone analysis. For each trial, 1 μL of pheromone sample solution was loaded. The retention time of the pheromone under the above-mentioned experimental conditions was noted to be ~5.2 min.

## Results and discussion

### Polyimide membrane deflection

**Figure 6:**
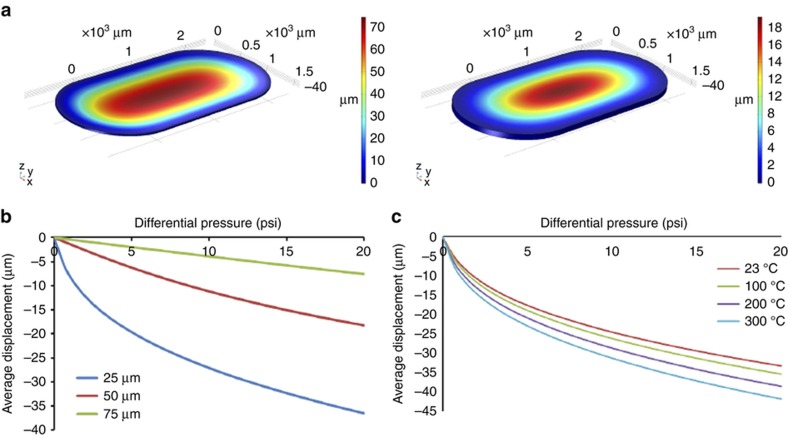
(**a**) Results simulated in COMSOL showing total displacement experienced by polyimide membrane under different thickness conditions. (**b**) Average displacement under different thickness conditions, with pressure ranging from 0 to 20 psi. (**c**) Average displacement under different temperature conditions, with pressure ranging from 0 to 20 psi.

The COMSOL simulation showed that the displacement of the membrane decreases with increasing membrane thickness, as illustrated in Figure 6a. Specifically, an increase in thickness from 25 to 75 μm results in a decrease in maximum displacement of the membrane from 70 to 20 μm, respectively. A similar trend was observed for the average displacement, which was calculated over the entire membrane region, as shown in Figure 6b. For a 25 μm-thick membrane, the average displacement was noted to be 35 μm, in contrast to a 75 μm-thick membrane, which can only achieve a maximum displacement of 5 μm under an applied pressure of 20 psi. Furthermore, we also observed a relatively greater displacement of the membrane when operated at elevated temperatures of 100, 200 and 300 °C compared to the one operated at room temperature (23 °C), as shown in Figure 6c. Specifically, the membrane experienced a 27% greater deflection at 300 °C compared to that at 23 °C. The average deflection of the membrane was noted to be 33 and 42 μm at 23 and 100 °C, respectively. This result occurred because the Young’s modulus of the membrane decreases as the temperature increases, which makes the polyimide membrane more flexible under elevated temperature conditions. The Young’s modulus of the polyimide membrane has been specified as being 3.1 GPa at 23 °C, 2.7 GPa at 100 °C, 2.2 GPa at 200 °C and 1.8 GPa at 300 °C (Ref. 38). Next, the Von Mises stress of the 25 μm-thick polyimide membrane was simulated under a uniform pressure of 20 psi, as shown in Supplementary Figure S5a. The largest stress experienced on the edge of the membrane was 8.2×10^7^ Pa, which is smaller than the yield strength (9×10^7^ Pa) of polyimide. Furthermore, the volumetric principal strain and first principal strain were also simulated under the same condition, as shown in Supplementary Figures S5b and c, respectively. The largest values of both strains are similar to the largest Von Mises stress. The largest values of strains at 20 psi pressure were 3%, being lower than the yield strength specified by the manufacturer of the polyimide membrane. Both the stress and strains analyses indicated that the 25 μm-thick polyimide membrane is safe to operate at 20 psi. Based on the simulation results, the thinnest polyimide membrane available (25 μm-thick membrane from DuPont Kapton HN) was selected to maximize the valve operation speed. Furthermore, a pure membrane without any coating (that is, Kapton HN) was selected to satisfy the high temperature and low gas adsorption requirements.

### Characterization of the leak rate

**Figure 7:**
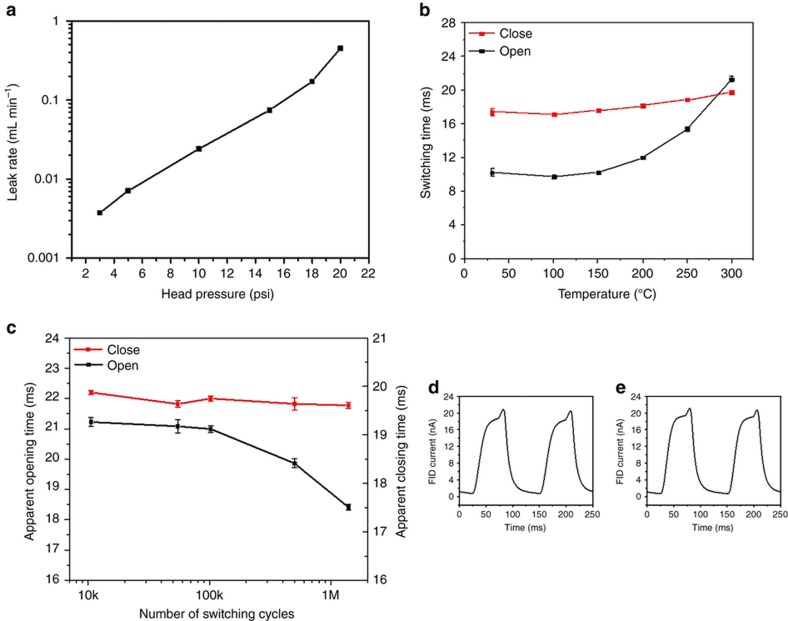
Characterization of the microelectromechanical systems (MEMS) flow control device. The channel separation *d* of the MEMS flow control devices was 500 μm. (**a**) Leak rate of the microvalve at different inlet pressures when the microvalve was closed by applying a pneumatic actuation pressure of 25 psi. (**b**) Apparent switching time of the microvalve at different temperatures up to 300 °C (*n*=6). (**c**) Reliability test of up to 1 million cycles at 300 °C (*n*=6). (**d**) Waveform of chopped hexane at 300 °C at the beginning of the reliability test. (**e**) Waveform of chopped hexane at 300 °C at the end of the reliability test.

The relationship between the leak rate and the inlet helium pressure was investigated using this system by increasing the head pressure from 3 to 20 psi, as shown in Figure 7a. It is evident from the graph that the leak rate increases with the increase in helium flow rate. Moreover, the open valve flow rate was measured using the same system by keeping one valve open and the others closed. For the microvalve under an inlet pressure of 5 psi, the lowest volumetric leak rate was measured to be ~7.6 μL min^−1^. The open valve flow rate was measured to be ~17.9 mL min^−1^. Thus, the on/off flow rate ratio of the valve was over 10^3^.

### Switching speed of flow control device

The switching speed of the valve was characterized by the apparent opening and closing times. The apparent opening time of the microvalve was defined as the time required by the FID signal to rise from 10 to 90% of the full amplitude value. Conversely, the apparent closing time was defined as the time required to drop from 90 to 10% of the full amplitude value. The switching speed of the flow control device was measured by chopping the hexane effluent at a frequency of 8 Hz and with a 50% duty cycle. The experimental result is shown in Supplementary Figure S4. The switching speed of the microvalve at different temperatures was investigated by changing the temperature from room temperature to 300 °C, as shown in Figure 7b. At room temperature, the apparent opening time and apparent closing time were 10.19±0.47 ms and 17.38±0.35 ms, respectively. As the temperature increased, both remained almost unchanged (the difference in apparent opening time was <5%, and that in apparent closing time was <2%) when the temperature remained below 150 °C. Beyond 150 °C, an increase in opening and closing times was observed. At 300 °C, the apparent opening time and apparent closing time were 21.30±0.30 ms and 19.80±0.04 ms, respectively. These times correspond to a 108.9% and 13.9% increase in comparison to the values at room temperature, respectively. The increase in the switching time is attributed to the increase in flexibility of the polyimide membrane at higher oven temperatures.

### Reliability of flow control device

For real MEMS-GC-EAG applications, the flow control device is required to switch at high temperatures for an extended number of cycles. The flow control device was allowed to switch continuously for more than 53 h, which corresponds to more than 1 million cycles for each microvalve. The microvalve remained fully functional, and in fact, even faster switching was observed at the end of the test. The apparent opening time and closing time dropped to 18.41±0.09 ms and 19.61±0.07 ms, respectively, which corresponded to decreases of 13.2% and 1.3%, respectively, as shown in Figure 7c. Comparison of the waveforms of the chopped hexane acquired before the beginning of or after the end of the reliability test at 300 °C confirmed that the waveforms were similar (Figures 7d and e). These results indicate that the microvalve successfully maintained its functionality during the reliability tests. Furthermore, no deterioration of its performance over a long period of operation was observed.

### Detection of pheromone using MEMS chopper-modulated GC-EAG

**Figure 8:**
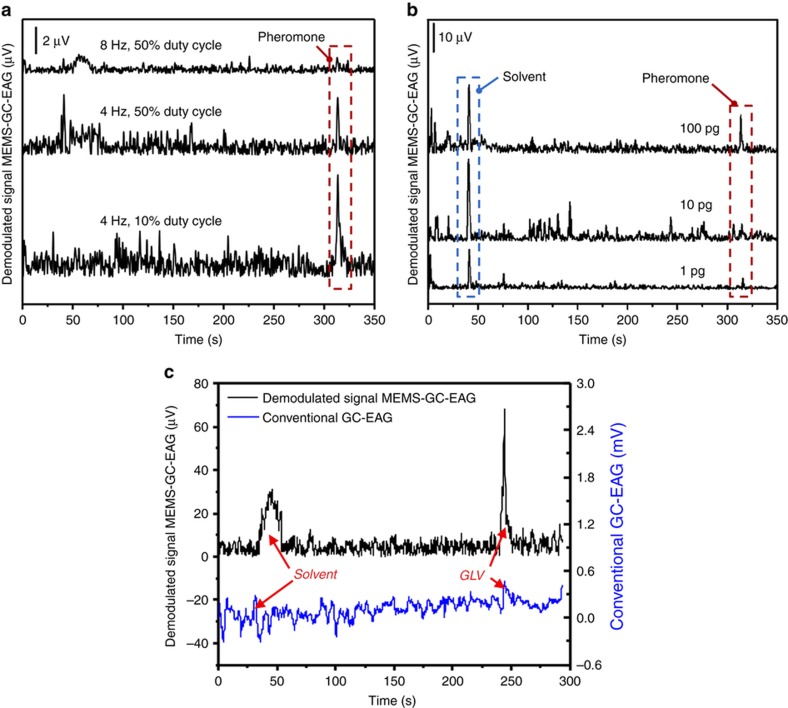
Use of microelectromechanical systems gas chromatography-electroantennography (MEMS-GC-EAG) for pheromone and other natural VOC detection. (**a**) Demodulated MEMS-GC-EAG signals acquired at different chopping frequencies and with different duty cycles. 10 ng of the *cis*-11-hexadecenal pheromone was loaded for each trial. (**b**) Demodulated MEMS-GC-EAG illustrating the dose response. The flow control device chopped the effluent at 2 Hz with a 10% duty cycle for each trial of *cis*-11-hexadecenal. (**c**) Comparison of the signal retrieved using the conventional GC-EAG and that retrieved using the MEMS-GC-EAG for GLV, a natural VOC. GLV, green leafy volatile; VOC, volatile organic compound.

Chopped GC effluent was expected to evoke waveforms modulated by frequency on the raw EAG recording. The antenna from *H. Virescens*, however, demonstrated a frequency- and duty-cycle-dependent response. It is clearly shown in Figure 8a that 10 ng of the *cis*-11-hexadecenal pheromone chopped at 8 Hz and with a 50% duty cycle is barely visible after the signal is demodulated. However, the peak signal can be easily detected when the frequency is decreased to 4 Hz for a similar duty cycle. Moreover, a 10% duty cycle produces a stronger signal than a 50% duty cycle at 4 Hz. In summary, the best results were obtained at 4 Hz with a 10% duty cycle, which is different from a previous report, where the best performance was achieved at 8 Hz for the *Helicoverpa zea* moth^26,31^. We believe that the condensation of pheromone occurs in the mixing tube, which is held at room temperature. When the device actually turns off its delivery of pheromone to the mixing tube, the condensed pheromone then evaporates into the mixing tube. Thus, continuous pheromone (instead of chopped pheromone) is delivered to the antenna. At a lower frequency and/or lower duty cycle, as the interval is comparatively longer, the condensed pheromone will evaporate from the tip of the transfer line before subsequent delivery of the pheromone to the mixing tube. This consideration is supported by the observation that as the interval between the potential peaks increases because of the decrease in chopping frequency, the characteristic pheromone signal of the chopped signal becomes more obvious in the raw EAG recordings, as shown in Supplementary Figure S6b. Furthermore, after demodulation, a 2 Hz, 10% duty cycle signal results in a higher pheromone peak than that of a 4 Hz, 10% duty cycle signal, as shown in Supplementary Figure S6a. However, increasing the interval by lowering the chopping frequency results in an elevated noise level, as shown in Figure 8a. A 2 Hz, 10% duty cycle was thus determined to be the optimal condition, and further experiments were carried out under this condition unless specified. Moreover, the dose response was also investigated by loading different amounts of pheromone into the GC injector. The absolute amplitude of the pheromone peak decreases as the dosage decreases, as shown in Figure 8b.

In addition to pheromone detection, we further investigated the advantage of MEMS-GC-EAG technology in detecting a generic, natural VOC, that is, green leafy volatile (GLV) (Z3-6 Ac). For this purpose, we employed antennae harvested from wild, black cutworm moths (*Agrotis ipsilonis*), as shown in Figure 8c. GLV was dissolved in hexane at a concentration of 1 μg μL^−1^. 1 μg of GLV could be detected using the MEMS-GC-EAG, chopping at 8 Hz and with a 50% duty cycle, with great certainty, whereas the same signal was hard to distinguish from the background noise using the conventional GC-EAG. The SNRs of the MEMS-GC-EAG and conventional GC-EAG were estimated to be 36.3 and 9.3 dB, respectively, which means that the SNR improvement of the MEMS-GC-EAG over the conventional GE-EAG in this case was ~22.4 times. It is interesting to note that each 1 μL GLV sample contains 1 μg of GLV and 654.80 μg of hexane. However, the amplitude of the GLV peak in the MEMS-GC-EAG was higher than that of the hexane solvent peak. This result can be attributed to the differential sensitivity of the sensory neuron to GLV rather than to hexane. The specificity of the sensory neuron with respect to some specific chemicals could be utilized to detect some specific chemicals that are at trace levels and hard to detect using the conventional methods. This augments our claim that in the near future, this enabling technology will permit various studies in the field of chemical ecology. As a reference, we also compared the detection capabilities of the conventional EAG and the FID for 1 ng of the Z11-16:Ald pheromone, as shown in Supplementary Figure S7. The SNRs of the EAG and the FID were estimated to be 22.8 and 8.6 dB, respectively. The superiority of the EAG as a detector over the conventional FID GC detector for pheromone detection is consistent with the literature^39^.

## Conclusions

The design, fabrication and characterization of a high-temperature flow control device have been demonstrated in this article. The device consists of a polyimide membrane bonded between two glass substrates. The device is packaged in an aluminum housing that interfaces inlets/outlets and peripheral parts used for temperature control and pneumatic control. The overall size of the device is relatively small (50 mm×50 mm), and the device can be installed inside most conventional GC ovens. The characterization of the device indicates that the flow control device can reliably switch at 300 °C for >1 million cycles. Moreover, we have also demonstrated that the on/off flow ratio of the microvalve is >10^3^.

Furthermore, an interesting application of the device, that is, enabling the MEMS-GC-EAG, has been demonstrated. Here, we first modulated the signal to a high frequency, followed by signal demodulation at the detector end. Since most GC-EAG devices have predominant noise sources at low frequencies, this modulation technique serves to improve the SNR of EAG detectors.
