# Supplementary material for: Chopper-modulated gas chromatography electroantennography enabled using high-temperature MEMS flow control device
Source: Microsyst Nanoeng. 2017 Dec 18;3:17062. doi: 10.1038/micronano.2017.62 (PMC6444993; doi:10.1038/micronano.2017.62)
Supplement: Supplementary Information [file micronano201762-s1.pdf]

Supplementary File

Chopper-modulated gas chromatography electroantennography enabled using high-temperature MEMS flow control device

Ming-Da Zhou[1,2,*], Muhammad Akbar[1,2,*], Andrew J Myrick[1,3], Yiqiu Xia[1,2], Waleed J Khan[1,4], Xiang Gao[1], Thomas C Baker[3] and Si-Yang Zheng[1,2,4]

Microsystems & Nanoengineering (2017) **3,** 17062; doi:10.1038/micronano.2017.62; Published online: 18 December 2017

1. JIG FOR BONDING MEMS FLOW CONTROL DEVICE

A bonding jig was designed and constructed for the bonding of the MEMS flow control device. The bonding jig could accommodate bonding of two devices simultaneously at elevated temperature as high as 450 °C (Supplementary Figure S1).

The jig consisted of two pieces of 25.4 mm-thick, 100 mm × 100 mm aluminium blocks, two pieces of 2 mm-thick, 28 mm × 28 mm aluminium pads, two pieces of 6.35 mm-thick, 50.8 mm × 50.8 mm square glass pads, and one pieces of 3.175 mm-thick, 50.8 mm × 50.8 mm square glass pad. The thickness of each piece in the bonding jig was carefully designed to compensate for the thermal expansion coefficient difference among them. All the glass pads were made of borosilicate glass unless otherwise specified. The jig was designed to apply uniform pressure across the whole area of the device during the bonding. Efforts were taken to make the surfaces of the metal part as flat as possible. The surface of the 25.4 mm thick aluminium block that faced towards chip was sanded flat after milling. Both surfaces of the 2 mm thick aluminium pad were sanded flat and the surface that faces the 6.35 mm-thick glass pad was further polished. 100 nm-thick aluminium film was deposited on one surface of the 6.35 mm-thick glass pad and both sides of the 3.175 mm-thick glass pads to prevent the undesired bonding between the glass substrate of the valve and the glass pad. The devices were aligned to the two 25.4 mm-thick aluminium blocks through four 0.8 mm-diameter alignment holes and alignment pins (McMaster-Carr, Cleveland, OH).

After assembling the devices with the bonding jigs, pressure was applied using stainless screws and torque wrench to ensure uniform pressure. After applying the pressure the alignment pins were removed.

2. TEMPERATURE CALIBRATION OF THE MEMS FLOW CONTROL DEVICE

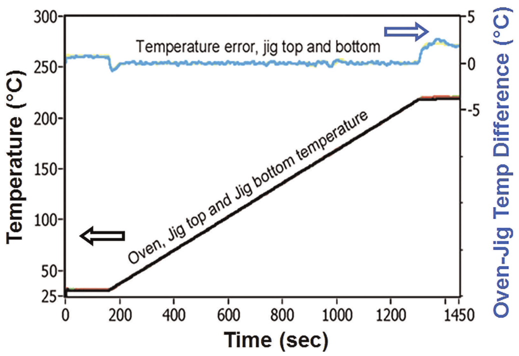

Figure S2 Temperature calibration of the MEMS flow control device showing the temperature profiles of the oven (black, bottom), the top jig (red, bottom) and bottom jig (green, bottom), as well as the temperature difference between the top jig (blue, top), bottom jig (yellow, top) and the oven.

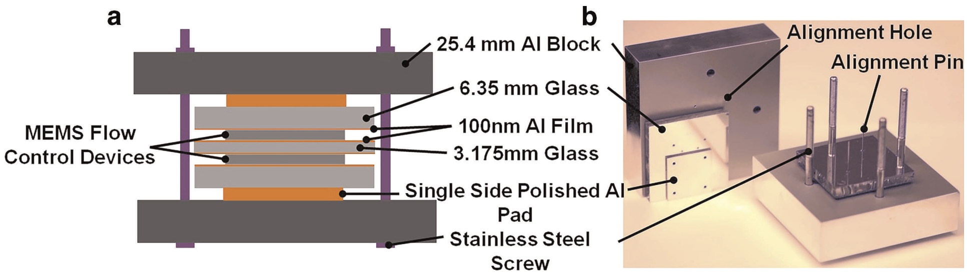

Figure S1 Custom-made bonding jig. (**a**) Cross-section schematic of the jig accommodating bonding of two MEMS flow control devices simultaneously. (**b**) Picture of the bonding jig with its components.

[1]Micro & Nano Integrated Biosystem (MINIBio) Laboratory, Department of Biomedical Engineering, The Pennsylvania State University, University Park, PA 16802, USA; [2]Materials Research Institute, The Pennsylvania State University, University Park, PA 16802, USA; [3]Department of Entomology, The Pennsylvania State University, University Park, PA 16802, USA and [4]Department of Electrical Engineering, The Pennsylvania State University, University Park, PA 16802, USA
Correspondence: Si-Yang Zheng (sxz10@psu.edu.)
[*]These authors contributed equally to this work.

3. CHARACTERIZATION OF THE LEAK RATE OF THE MEMS FLOW CONTROL DEVICE

To set up the glass vial for the leak rate measurement of the microvalve, a 5 mm-diameter hole was drilled in the cap of the vial and a piece of PDMS (Sylgard 184 Silicon Elastomer Kit; Dow Corning, Midland MI) gasket[1] with diameter matching the openings of the vial and the cap was prepared. The vial was filled full with deionized (DI) water and the PDMS gasket was sandwiched between the cap and the opening of the vial to seal the vial. A 0.32 mm-I.D. capillary column was inserted into the vial through the PDMS gasket and served as inlet. Another piece of 0.53 mm-I.D. capillary column was also inserted into the vial through the PDMS gasket and served as the outlet. The vial was held upside down so that any water that was displaced by the gas leaking through the on-chip microvalve would escape the vial through the vent and generate a weight loss.

4. CHARACTERIZATION OF THE SWITCHING SPEED OF THE MEMS FLOW CONTROL DEVICE

The switching speed of the device was measured by chopping hexane, a commonly used solvent in GC separation, at 8 Hz and a duty cycle of 50%. The device was set to chopping operation prior to the loading of hexane. 1 μL hexane was loaded into the injector. Due to the abundance of hexane as a solvent, it was always inevitably treated as overloaded in this experiment. Thus its characteristic peaks saturated the FID and had an undesirable expansion. Due to the overloading of hexane, a fair amount of residual would remain in the column. The switching speed was acquired from chopping the effluent of the hexane residual, which did not saturate the FID (Supplementary Figure S4).

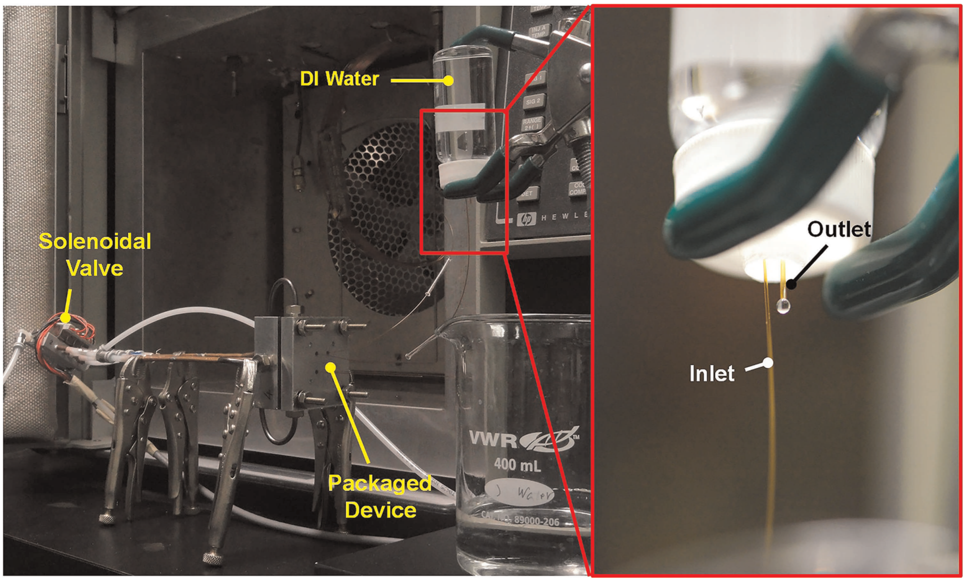

Figure S3 Experimental setup for the leak rate test. Experimental setup for the leak rate test. A simplified setup, which did not have temperature control unit, was employed to provide connection interface for the MEMS flow control chip to the pneumatic control lines and the capillary column, which was connected the injector of the GC system.

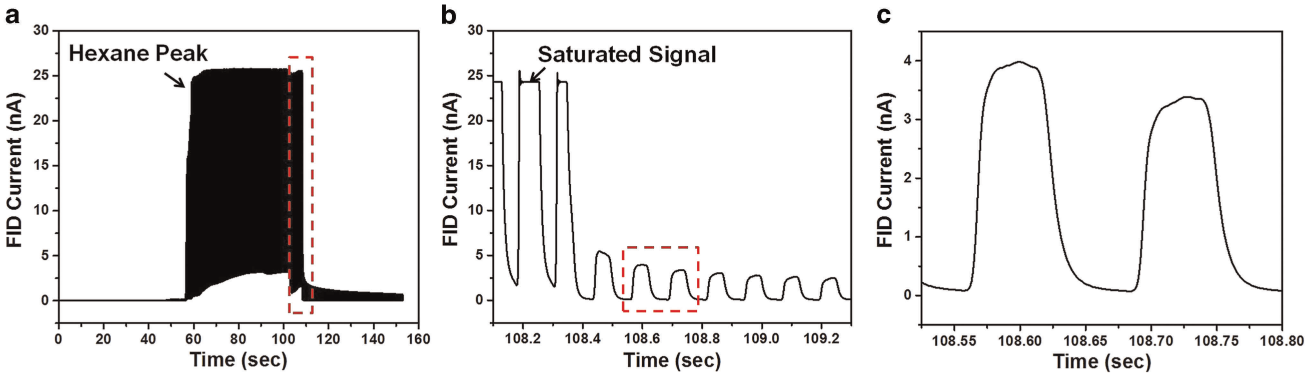

Figure S4 Chopping of hexane signal at 8 Hz, 50% duty cycle at room temperature. (**a**) Characteristic hexane peaks saturated the FID and had an undesirable expansion due to the overloading. (**b**) Close-up view of the waveforms that saturated the FID and the first several waveforms that did not saturate the FID (the dashed rectangle in **a**. (**c**) Close-up view of the waveforms that did not saturate FID (the dashed rectangle in **b**. Switching speed was acquired from the waveforms that did not saturate the FID.

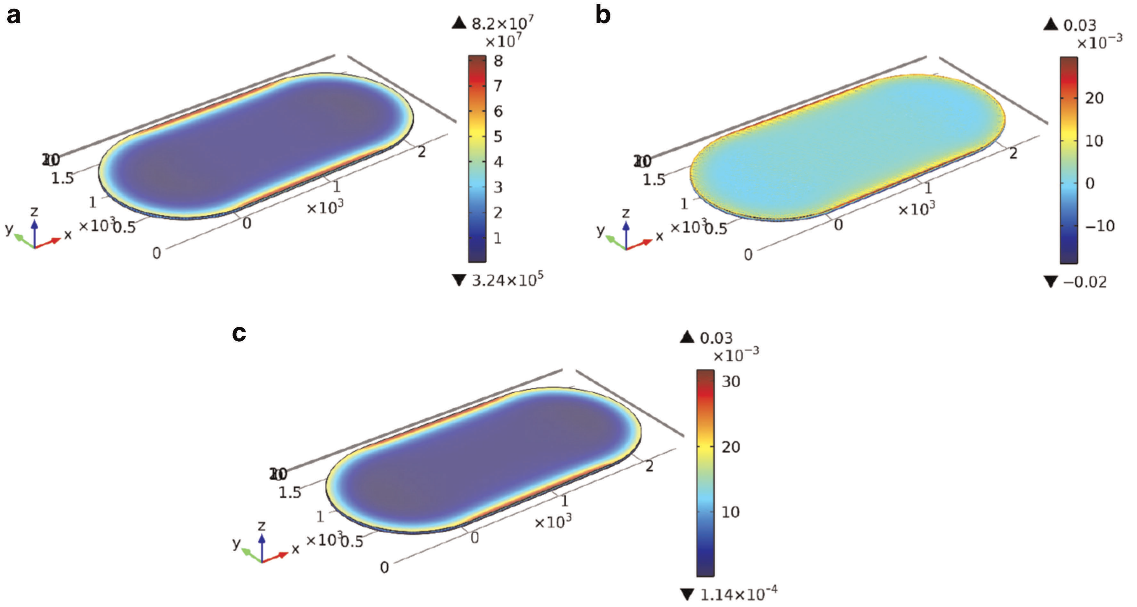

Figure S5 Stress and strain analysis of polyimide membrane simulated in COMSOL. (**a**) Von Mises stress (Pa). (**b**) Volumetric strain. (**c**) First principal strain.

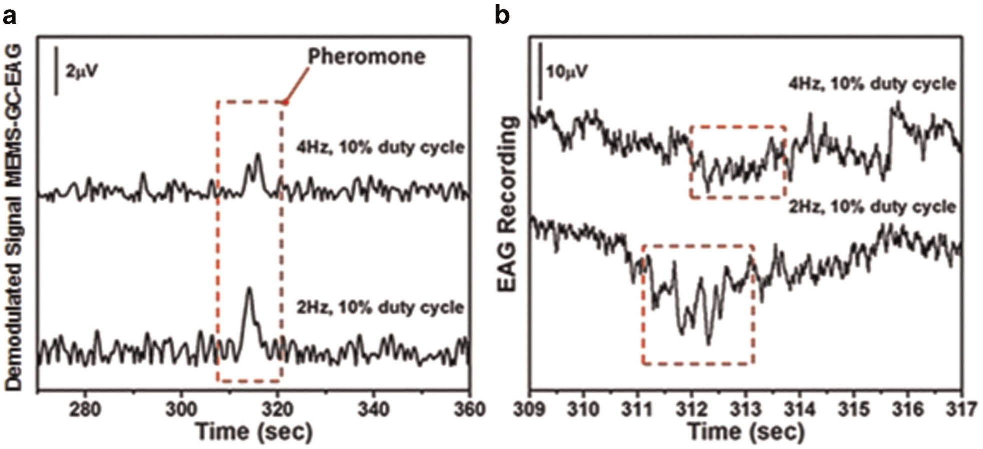

Figure S6 Frequency response of the *H. Virescens* antenna to pheromone *cis-11-hexadecenal* at chopping frequencies of 2 and 4 Hz. 1 ng pheromone was loaded for each trial. Both trials were performed with the same antenna. (**a**) Demodulated MEMS-GC-EAG signals. Pheromone peaks are indicated with a dash rectangle. (**b**) Raw EAG recordings. Signals evoked by pheromone chopped at different frequency are indicated with dash rectangles.

5. STRESS AND STRAIN ANALYSIS OF THE POLYIMIDE MEMBRANE

The COMSOL simulation model for stain analysis was set up by selecting 3D as the space dimension, electromechanics as the physics interface and stationary from the preset study list. The dimensions of the membrane are the same as the actuation chamber in the flow control device (two 1.6 mm diameter halfcircles separated by a square of 1.6 mm by 1.6 mm). Next, the boundary conditions for the membrane were defined by restricting the movement of the membrane edges to zero in all directions. The properties of polyimide membrane were specified in terms of Young's modulus of 3.1 GPa, density 1420 kg m^{-3} and Poisson's ratio 0.34. Within the study node, 20 psi was set to get the largest stress and strain of the membrane, since it is the largest potential pressure loading in our study. Von Mises strain, first principal stress and volumetric stress were analyzed as.

6. COMPARISON OF MEMS-GC-EAG PHEROMONE RESPONSES AT DIFFERENT MODULATION FREQUENCIES

7. COMPARISON OF EAG RECORDING WITH FID RECORDING IN A CONVENTIONAL GC-EAG SETUP

Using techniques outlined in[2], the SNR of the EAG and FID are estimated to be 22.8 dB and 8.6 dB, respectively. The EAG has been filtered with a first order high pass, corner frequency of 0.1 Hz and a 5th order Bessel lowpass filter with a corner frequency of 0.5 Hz. The FID output has also been filtered with an

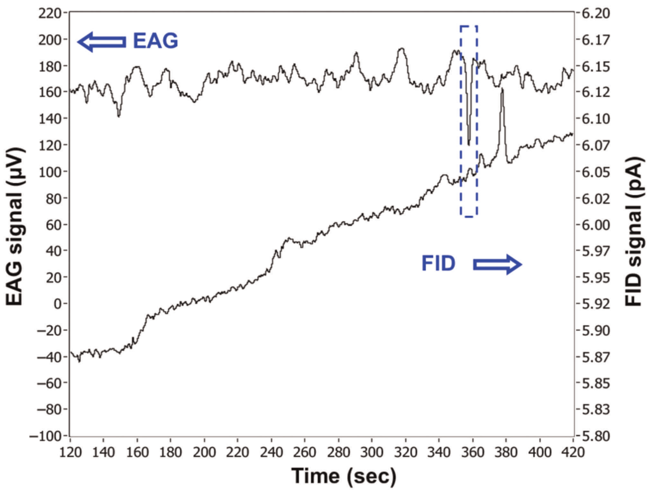

Figure S7 Simultaneous recording of GC-EAG and FID responses to approximately 6 pg of Z11-16:Ald.

identical Bessel low-pass filter. Its rising slope is the result of increased column bleed as the oven temperature is increased. Because the presence of some contaminants in the sample are evident on the FID, the vertical rectangle highlights the responses in both recordings due to the elution of Z11–16:Ald. The GC parameters are the same with two exceptions. Rather than using a splitless injection,a split ratio of 100:1 and a 1 ng quantity of Z11–16:Ald was utilized to reduce contaminating peaks. The column employed was a DB-1.

COMPETING INTERESTS

The authors declare no conflict of interest.

REFERENCES

1 Xia YN, Whitesides GM. Soft lithography. *Annual Review of Biomedical Engineering* 1998; **28**: 153–184.

2 Myrick AJ, Baker TC. Chopper-modulated locked in amplified gas chromatography—electroantennography Part II: Signal processing and performance comparisons. *IEEE Sensors Journal.* 2012; **12**: 2974–2983.
